# Supplementary material for: Towards Digital Twin Implementation in Roll-To-Roll Gravure Printed Electronics: Overlay Printing Registration Error Prediction Based on Printing Process Parameters
Source: Nanomaterials (Basel). 2023 Mar 10;13(6):1008. doi: 10.3390/nano13061008 (PMC10053699; doi:10.3390/nano13061008)
Supplement: Supplementary file 1 [file nanomaterials-13-01008-s001.zip › nanomaterials-2243133-supplementary.pdf]

Supplementary Materials

Towards Digital Twin Implementation in Roll-To-Roll Gravure Printed Electronics: Overlay Printing Registration Error Prediction Based on Printing Process Parameters

Anood Shakeel [1,†], Bijendra Bishow Maskey [2,†], Sagar Shrestha [1], Sajjan Parajuli [2], Younsu Jung [2] and Gyoujin Cho [1,2,*]

[1]Department of Intelligent Precision Healthcare Convergence, Sungkyunkwan University, Suwon-si 16419, Republic of Korea; anood.shakil@g.skku.edu (A.S.); sagar@g.skku.edu (S.S.)
[2]Department of Biophysics, Institute of Quantum Biophysics, Research Engineering Center for R2R printed Flexible Computer, Sungkyunkwan University, Suwon-si 16419, Republic of Korea, bizen@g.skku.edu (B.B.M); sajjanprj@gmail.com (S.P.); isinu7@skku.edu (Y.J.)
* Correspondence: gcho1004@skku.edu
† These authors contributed equally to this work.

Supplementary Figures

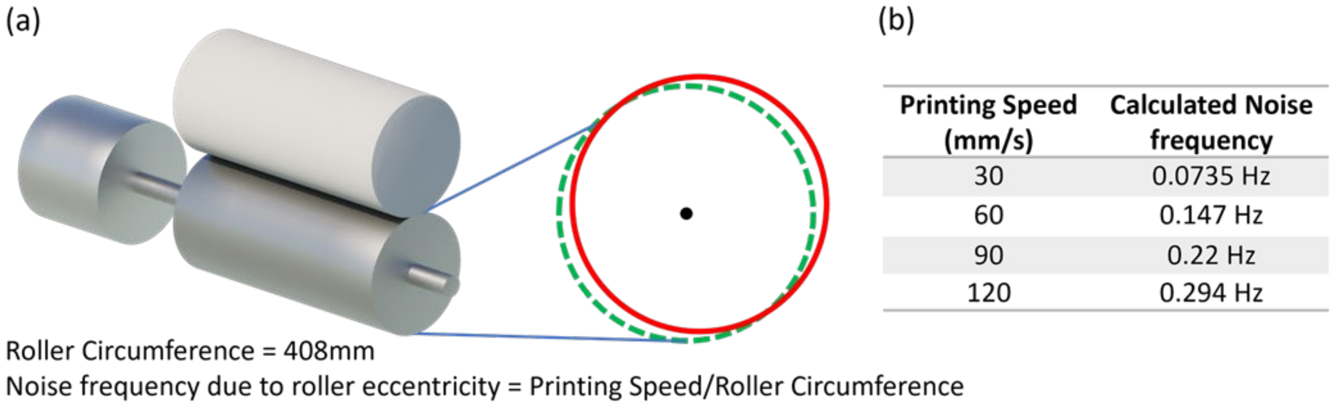

Figure S1. (**a**) Schematic showing gravure roller eccentricity resulting in nip force fluctuation, (**b**) noise frequency due to roller eccentricity calculated considering roller circumference of 408 mm at different printing speed.

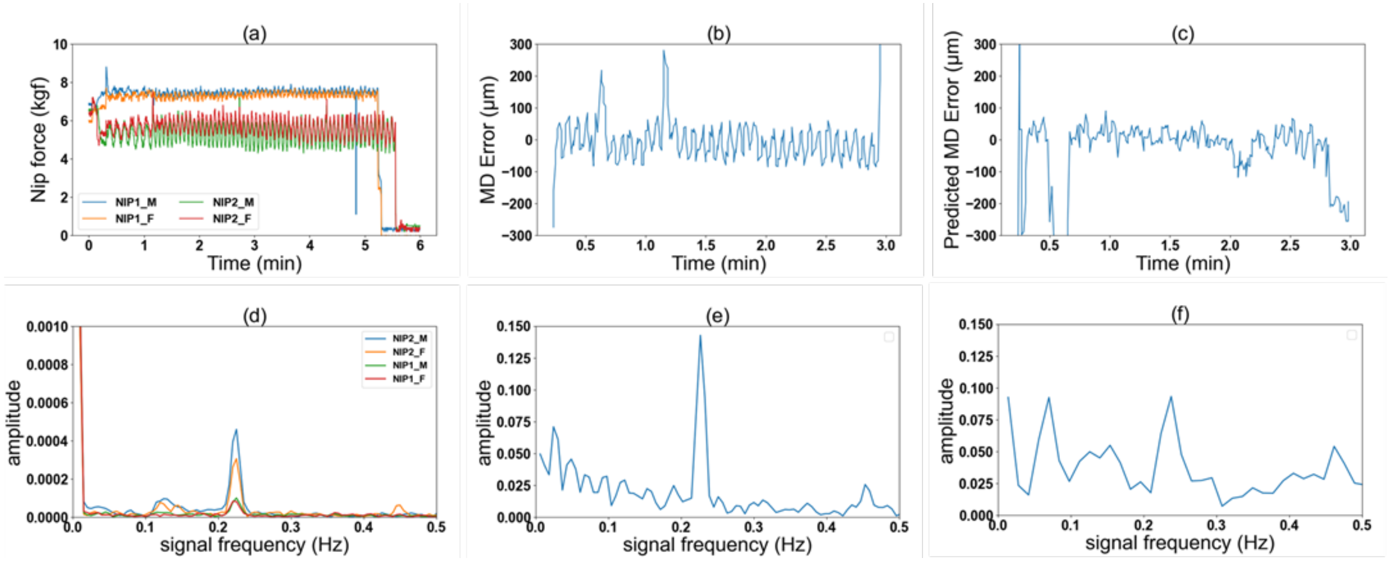

Figure S2. Time domain data of (**a**) nip pressure, (**b**) observed overlay error in MD, and (**c**) predicted overlay error in MD. Frequency domain data of (**d**) nip pressure, (**e**) observed overlay error in MD, and (**f**) predicted overlay error in MD.

Supplementary Tables

Table S1. Description for the typical parameters in the R2R gravure process in Figure 1.

Parameter	Description	Parameter	Description
T1	tension in the unwinder	NIP1_M	nip force in the first unit (motor side)
T2 and T3	tension in the first unit	NIP1_F	nip force in the first unit (front side)
T4 and T5	tension in the second unit	NIP2_M	nip force in the second unit (motor side)
T6	tension in the out feeder	NIP2_F	nip force in the second unit (front side)

Table S2. Data mapping algorithm for registration error, tension and nip force.

Cases	Camera triggers	Tension and nip force data
Case 0	0 trigger	5 data points / s -> 0
Case 1	1 trigger	5 data points / s -> 1
Case 2	2 triggers	5 data points / s -> 2
Case 3	3 triggers	5 data points / s -> 3

Table S3. Tension and nip force data.

Timestamp	T1	T2	T3	T4	T5	T6	NIP1_M	NIP1_F	NIP2_M	NIP2_F
2022-10-05 12:24:20.0	5.9	5.2	5	4.7	4.5	3.9	3.8	4.5	5.9	5
2022-10-05 12:24:20.2	5.5	5.3	5	4.7	4.4	3.95	3.9	4.4	5.8	5.1
2022-10-05 12:24:20.4	5.5	5.4	5	4.6	4.3	3.9	3.9	4.3	6	5.2
2022-10-05 12:24:20.6	5.9	5.4	5	4.7	4.3	3.85	3.9	4.4	6.1	5.4
2022-10-05 12:24:20.8	5.5	5.6	5	4.8	4.3	3.85	4.2	4.7	6.3	5.7
2022-10-05 12:24:21.0	5.8	5.6	4.9	4.7	4.5	3.9	4.4	4.7	6.4	5.8
2022-10-05 12:24:21.2	5.6	5.6	4.9	5	4.6	3.95	4.7	4.9	6.4	5.6
2022-10-05 12:24:21.4	5.5	5.5	4.9	4.9	4.5	4	4.7	5.1	6.3	5.6
2022-10-05 12:24:21.6	5.4	5.5	4.9	4.9	4.5	3.95	4.8	5.4	6.2	5.5
2022-10-05 12:24:21.8	5.4	5.4	4.8	5	4.5	4	5.1	5.5	6.2	5.3
2022-10-05 12:24:22.0	5.4	5.3	4.9	4.7	4.3	4.05	5.5	5.7	6.2	5.1
2022-10-05 12:24:22.2	5.4	5.3	4.9	4.8	4.3	4.05	5.6	5.8	6.2	5
2022-10-05 12:24:22.4	5.4	5.4	4.9	4.6	4.3	4.05	5.9	5.8	6.2	5
2022-10-05 12:24:22.6	5.4	5.4	4.9	4.7	4.3	4	5.8	5.8	6.2	5.1

2022-10-05 12:24:22.8	5.8	5.4	4.8	4.7	4.3	3.9	5.7	5.6	6	4.9

Table S4. MD and TD camera triggers data.

Day	Time	Minute	Second	Film_Result_X [mm]	Film_Result_Y [mm]
5	12	24	9	0.196	1.183
5	12	24	9	0.083	1.139
5	12	24	10	–0.036	0.792
5	12	24	10	–0.062	0.444
5	12	24	11	–0.037	0.115
5	12	24	12	0	–0.135
5	12	24	12	0.013	–0.347
...	…	…	…	…	…
5	12	27	52	–0.012	–0.605
5	12	27	52	–0.021	–0.562
5	12	27	53	0.02	–0.534
5	12	27	53	–0.007	–0.547
5	12	27	54	0	0

Table S5. Mapping tension and nip force data to MD and TD camera triggers data.

Timestamp	Film_Result_X [mm]	Film_Result_Y [mm]	T1	T2	T3	T4	T5	T6	NIP1_M	NIP1_F	NIP2_M	NIP2_F
…												
2022-10-05 12:25:00	0	–0.49	6	5.2	5	4.6	4.2	4	5.5	5.3	5.8	4.7
2022-10-05 12:25:00	–0.006	–0.479	6	5.3	5	4.6	4.2	4.05	5.1	5.2	5.7	4.7
2022-10-05 12:25:01	–0.007	–0.514	5.88	5.38	5	4.66	4.44	3.88	4.1	4.7	5.8	4.76
2022-10-05 12:25:02	–0.011	–0.537	5.73	5.23	5	4.6	4.3	3.92	3.53	4.07	5.83	4.97
2022-10-05 12:25:02	–0.014	–0.604	5.65	5.2	5	4.55	4.3	3.925	4	4.4	6.05	5.25
2022-10-05 12:25:03	–0.014	–0.578	5.5	5.37	4.97	4.7	4.37	3.9	4.33	4.77	6.37	5.73
2022-10-05 12:25:03	0.007	–0.552	5.4	5.4	5	4.65	4.5	3.85	5.1	5.4	6.3	5.5
2022-10-05 12:25:04	0.017	–0.532	5.57	5.27	4.97	4.57	4.43	3.9	5.63	5.77	6.3	5.3
2022-10-05 12:25:04	0.006	–0.479	5.75	5.2	5	4.6	4.3	4	5.7	5.85	6	4.9
2022-10-05 12:25:05	0	–0.481	5.72	5.36	5	4.68	4.34	3.86	4.8	5.2	5.78	4.74
